# Advances and challenges in the use of artificial intelligence for the diagnosis of osteoarthritis

**DOI:** 10.1590/0100-3984.2023.56.5e4-en

**Published:** 2023

**Authors:** Júlio Nather Junior

**Affiliations:** 1 Radiologist at the Clínica CEDIRP and at the Hospital das Clínicas da Faculdade de Medicina de Ribeirão Preto da Universidade de São Paulo (HCFMRP-USP); Technical Coordinator of the HCFMRP-USP Center for Innovation and Technology, Ribeirão Preto, SP, Brazil. julio.nather@gmail.com.

Technological development has been a fundamental driver in health care, particularly in the field of radiology, in which an accurate diagnosis is crucial for instituting effective treatment. The use of convolutional neural networks (CNNs) for the radiographic analysis of knee osteoarthritis, as described in a study published in this issue of Radiologia Brasileira^(^1^)^, is a transformative step that promises to facilitate the identification of knee osteoarthritis, which is a prevalent and debilitating condition. That study presents a CNN model developed specifically to diagnose knee osteoarthritis based on radiographs. The innovation lies in the application of a computational model trained with an extensive local dataset, potentially overcoming the barriers of variability in clinical presentation and radiographic image quality that are inherent to the populations studied previously.

A CNN represents the state of the art in the processing and analysis of medical images, offering an approach that does not require the manual selection of relevant attributes^(^2^)^. The model proposed in the study in question^(^1^)^ constitutes a considerable advance, using a densely connected architecture that allows full use of the information extracted from images, which is crucial for diagnostic accuracy. However, the limitations inherent to this approach cannot be ignored. One of the most critical issues is the need for large volumes of annotated data to train these systems, which poses logistical challenges and raises privacy concerns. In addition, the potential bias introduced by the fact that the population involved was from a single center could limit the generalizability of the results to other demographic groups. Finally, although CNNs reduce the workload of radiologists, the interpretation of their output is still essential to ensure the accuracy and reliability of the diagnosis, which underscores the complementarity between the machine and the human expert.

Despite the limitations mentioned above, the use of CNNs in the radiographic diagnosis of knee osteoarthritis is promising^(^3^,^4^)^. The integration of such technologies into the health care system could provide a significant improvement in diagnostic accuracy, reducing costs and optimizing the workflow of health care professionals. The model presented in the article in focus^(^1^)^ suggests a way to move in that direction, while recognizing the need for external validation and continuous improvement of the system.

The current state of research in this area indicates that we are on the threshold of a new era in diagnostic radiology, driven by the development and integration of advanced artificial intelligence techniques^(^5^)^. Although artificial intelligence will not replace human clinical judgment and experience, it is establishing itself as a powerful tool for expanding diagnostic capabilities, opening new avenues for patient care, and charting the future of personalized medicine. The CNN model outlined in this recent study^(^1^)^ is an inspiring example of that future, a step toward harmony between the use of artificial intelligence and human health care.
